# On Iodide of Potassium

**Published:** 1854-01

**Authors:** 


*On Iodide of Potassium.* By J. W. Corson, M. D. Cases testing the Iodide of Potassium, as an antidoed to the injurious effects of Mercury, and corroborative of the experiment of M. Melsens, (read before the Medical Society of the N. Y. Dispensary.)

Case I.—*Remittent Fever; Hepatic Congestion; Anasarca; Recovery.* Mr.——, æt. 35, trunk-maker, slender, pale, having previously suffered from a chronic cough, suspected to be phthisical, was under my care during a severe attack of bilious remittent fever, in September, 1842.

With other febrile symptoms, there was a slight jaundiced hue of the skin and conjunctiva, a yellow fur upon the tongue, unusual thirst, nausea and irritability of the stomach, highly-colored urine, a teazing, dry cough, unaccompanied by any appreciable lesion of the lungs, but with decided tenderness *over the region of the liver.* He was ordered cooling diaphoretics, warm sponging, and grain and a half doses of calomel, with Dover's powder, every four hours, until, more readily than usual, very severe and protracted ptyalism supervened. His recovery was tedious, and some very free lay discussions on the treatment of the "Calomel Doctor," gave the case unusual interest. Under the use of quinine and bitter infusions, he rallied feebly, but the hepatic tenderness and jaundice remained for some time, and he finally relapsed with extensive anasarca.

In consultation with a medical friend, it was decided not to push the mercurial farther in an apparently strumous habit, but to trust to counter-irritation, and the exhibition of five grains of iodide of potassium three times a day. The dropsical effusion soon after disappeared; the remedy was continued for several weeks after, till recovery was perfect.

He has since enjoyed improved health, and has suffered no permanent ill effects from the mercurial treatment.

CASE II.—*Obstructive Ulceration of Throat; severe Ptyalism; Tedious Recovery.*—Mrs. ——, æt. 30, dress-maker, of good character, pale, delicate, and of a strumous family, was placed under my care in Nov., 1843, with extensive ulceration of the throat, of two years' standing, involving principally the pharynx and upper portion of the œsophagus, and so severe as to prevent her from swallowing solids, for months, and to limit her to soups and liquid food, and incapacitate her, and confine her to her room. Her appetite was poor; the menses were suppressed, and her spirits prostrated with the apathy of despair.

The fauces were red and extensively ulcerated; the teeth quite blackened, to use her expression, with the "powerful medicine" of my predecessors, and the profuse flow of saliva, the fetid breath and spungy gums, indicated that she was suffering from mercurial ptyalism. A strong solution of the nitrate of silver, acid gargles, and the usual topical remedies, had already been faithfully tried.

With slight hopes, I kept her upon five grains of the iodide of potassium three times a day, with rich broths and gentle exercise, and after several weeks, slight amendment was perceived. The granulations very slowly healed; her strength returned, and she was at last enabled to take long walks, and swallow and digest beef-steak and other solid food. She continued to take the iodide of potassium, in five-grain doses, three times a day, with slow, but visible improvement, and with little intermission for *eleven months.*

She entirely recovered; resumed her occupation, and, till lost sight of, some three years since, continued in good health, suffering none of the constitutional ill effects of mercury.

CASE III.—*Pleuritic Effusion; Displacement of the Heart; Gradual Recovery.*—Mr. ——, æt. 36, shoe-dealer, slender, and of a scrofulous family, having been exposed to cold, was seized in December, 1851, with excruciating pain in the left side of the chest, accompanied with great difficulty of breathing, dry cough, fever, and a hard, bounding pulse.

In spite of prompt bleeding from the arm to twenty ounces, and full doses of antimony, followed by cupping and hydragogue purgatives, and blisters to the side, effusion soon supervened, accompanied by the usual physical signs, viz: want of motion, bulging, flattened intercostal spaces, dullness on percussion, and absent respiration.

The heart was finally pushed to right of the sternum.

Notwithstanding the strumous taint, I was at length obliged to resort to a mercurial course, and trust to correct its injurious effects by the subsequent use of iodide of potassium. With free mercurial ptyalism, absorption soon commenced. It was continued for many weeks, and with the use of a succession of blisters and the iodide of potassium, with the extract of conium, till the following spring. The effusion was completely absorbed, the heart returned to its normal position, and the patient escaped with slight contraction of the side affected. He continues in his usual good health.

CASE IV.—*Suffocative Laryngitis; Ptyalism; suppression of Urine; Recovery.*—Mrs. ——, æt. 40, respectable, of good constitution, having been exposed to cold, was seized in October last with chilliness, succeeded by hoarseness, cough, sense of constriction, tense, quick pulse, and fever, which increased, till in a few hours the face grew dusky, the features anxious, and she seemed ready to suffocate with acute larynigtis.

It was necessary to be in constant attendance upon her, and I resorted promptly to emetics, leeches to the top of the sternum, with antimonials, warm inhalations, and three grains of calomel and five of Dover's powder every three hours, till the breathing grew easier, the pulse softer, and the face more tranquil. Two days after, she began to flag a little; the pulse grew small and soft, and she had turns of faintness, accompanied by a sort of hysterical panting and choking, evidently nervous and spasmodic, which were relieved by draughts of carbonate ammonia, with tinct. valerian and hyoscyamus. In a few hours the breath grew fetid, the gums tender, and she was seized with most profuse and painful ptyalism. From this time the dispnœa, cough, and laryngeal symptoms abated, while the prostration and nervousness continued. She was really a most pitiable object. The tongue was largely swollen, and indented in its sides, the cheeks were extensively excoriated and tender, and the saliva ran profusely down the chin. She could neither eat, drink or speak, for pain, and conversed by writing on a slate. Even the assurance of a thoughtful consulting medical friend, and my own, that the sore mouth had probably saved her life, seemed small comfort. After trying in vain mild saline purgings, opiates, free ventilation, astringent and sedative gargles, and powdered sulphur, I succeeded in mitigating her sufferings a little by carefully brushing over the ulcerated spots on the tongue and cheeks with a forty grain solution of the nitrate of silver by means of a camel's hair brush, coating them immediately after with a brush dipped in olive oil and laudanum. She had been subject, for years, to turns of dull pain about the kidneys, nausea, and excruciating headaches, (possibly from the retention of urea,) and a very scanty secretion of urine, and this latter symptom became very serious during the present attack. To relieve the ptyalism, and produce a mild diuretic effect, she was ordered five grains of the iodide of potassium in solution, with an equal quantity of the extract of taraxacum, and two grains of the extract of conium three times a day. The effect was delightful; the mouth, rapidly healed; the urinary secretion increased; the nervous symptoms abated; in a few days she regained her lost strength and appetite, and has been since that period, to use her own expression, "better than she has been for years." As a sure ofsafety, she continued to take the iodide of potassium for nearly a month.

Case V.—*Puerperal Fever; Severe Ptyalism; Recovery.*—Mrs. ——, æt. 35, dress maker, with blue eyes, light strumous complexion, of a phthisical family, and subject to chronic cough, was safely confined of her fifth child by a rapid, easy labor, early in January last. The after-pains were rather severe, but she did otherwise well till the evening of the sixth day, when I was suddenly summoned to relieve a somewhat severe abdominal pain, tenderness on pressure, with headache and some fever, which had been preceded by chilliness; the tongue be ng moist, the lochia and milk still free, and the pulse not much accelerated. She was not bled, but simply ordered warm fomentations, and fifteen grains each of calomel and Dover's powder, to be followed by a purge of castor oil, and oil of turpentine in the morning. She slept tolerably, and the purgative operated very freely, producing some temporary relief. She had suffered a teazing cough some time, had recently lost two sisters with consumption, and I was unwilling to salivate. Sinapisms were freely applied, and she was directed to take half a teaspoonful of oil of turpentine in sweetennd mucilage, with two-and-a-half-grain doses of opium every four hours. The uterus being enlarged and tender, and some umbilical pain remaining next day, eight leeches were applied. On the fourth day of the attack, all the symptoms became aggravated; the lochia ceased, the milk rapidly diminished, the pulse rose to 130, and became small and wiry; the features anxious and sharp; the pain and tenderness were more acute. She was immeditaely bled from a large orifice twenty ounces, and put upon two and a half grains each of calomel and opium every three hours.

The pulse grew softer and slower, the features more calm, and the pain soon ceased to disturb. She started in a sort of stupor occasionally during the night, and muttered a few incoherent sentences, apparently from the full opiate, but otherwise was much quieter. She continued thus until the second day after, when profuse ptyalism occurred, all her symptoms improved, and her entire attention was diverted to her mouth. I lost no time in resorting to the local treatment mentioned in the former case; penciled the ulcered tongue and cheeks with a forty grain solution of the nitrate of silver, followed immediately with olive oil, washing the mouth at intervals with a weak gargle of extract of conium, and tanin and honey dissolved in water. I commenced immediately, as a *remedy for the ptyalism*, and an antidote to the dreaded constitutional effects of mercury in a strumous habit, to give five grains of the iodide of potassium with two grains of the extract of conium three times a day. She rapidly recovered; the teazing cough ceased, and she continued the medicine for a few weeks, accompanied by porter and beef-steak, which she discussed with a ferocious appetite, and soon permanently regained her lost strength.

*Remarks.*—In common, doubtless with many others, I have for years been in the habit, especially in scrofulous constitutions, of always terminating a mercurial course by the protracted administration of the iodide of potassium, both as a remedy for the immediate effects of mercury, and as a prophylactic against its future injurious consequences. And I have gradually ventured to be far less apprehensive of the ill effects of the mercurials in cachectic habits, when judiciously managed, than formerly.

These views came as quiet, practical teachings from the bedside. But the peculiar mode of action by which the iodide of potassium neutralized the slow poison of mercury in the system, was to me, as doubtless to others, a mystery, till the recent clear and satisfactory experiments of M. Melsens, of Paris, explained the whole matter. The observations of M. Melsens were first embodied in a remarkably interesting paper, published not long since in the *Annales de Chimie et de Phisique*, and recently translated by Dr. Budd, of Bristol, and republished in the *British and Foreign Medico-Chirurgical Review* for January, 1853. Those who are not already familiar with it we would refer to the original paper, as a master piece of medical observation, strengthening the patient deductions from the experience of the bedside, by the most rigid chemical analysis.

In the hope of directing more attention to these researches, and somewhat confirming them, we have selected the above cases out of many others that might have been quoted. Some of them have the advantage of having been watched for several years, during which time they have exhibited no wondering pains, or undue sensitiveness to cold, and no cachetic appearance or other signs of injury from the constitutional effects of mercury. Four out of the five cases had legitimate evidence of scrofulous or tuberculous taint, and were, therefore, specially liable to injury, and better proofs of the efficacy of the antidote. In the single exception, too, *case No. 1 V*, there was a chronic renal affection, and it is well known that in Bright's disease, and other chronic difficulties of the kidney, mercury is particularly apt to act at times with fearful violence. The suffering in this case from ptyalism, was most severe of all, and the prostration amounted almost to that of mercurial erethism.

Yet, with these constitutional tendencies to hinder us in the administration, they all belonged to that desperate class of cases in which, from change of structure, or effusion of lymph or serum, life itself was immediately jeopardized, and apparently could only be saved by the resolvent effects of mercury. The simple enumeration of the cases would tell the practical physician that, when menacing life, he had, in duty to his patient, no other choice. They consist, it will be recollected, of extensive dropsy with hepatic congestion, threatening obstructive inflammation of the throat, immense pleuritic effusion, suffocative laryngitis, and severe puerperal paritonitis. In all these cases mercury appeared the *only remedy* capable of meeting the exigency. It succeeded most happily; and through the singular, and as we firmly believe, efficient, counteracting agency of the iodide of potassium, in a class of subjects most liable to injury, it left no sting behind. How the iodide of potassium acts in thus neutralizing the slow poison of mercury, the varied experiments of M. Melsens seems clearly to explain.

He lays it down as an admitted principle, proved by the well-known fact, that years afterward persons who have once freely taken mercury find gold coins discolored by the mercurial in the perspiration of their bodies, and that mercury has been sometimes detected in the body after death, *that mercury as well as lead combines with the animal tissues*, and remains, so to speak, fixed in the system for years. Secondly, that in the body as well as out of the body, the iodide of potassium acts as a *powerful solvent to the compounds of mercury and lead; disengages them readily from the animal tissues*, and drains them off through the kidneys. Many ingenious chemical and clinical experiments are given to establish these propositions. M. Melsens first proved that the iodide of potassium passes off principally in the urine, by taking large quantities himself, and then analyzing the different secretions of the body. The fæces contained scarcely a trace, while the urine was loaded with it. It passes off through the kidneys with great rapidity. A person took 77 grains of the iodide of potassium, and in a few minutes the urine was charged with it. The compounds of mercury and lead, with the iodide of potassium, pass off by the kidneys in the same way.

An extraordinary cure of a looking-glass maker, with severe mercurial paralysis, is given, in which the patient took the iodide of potassium in very large doses for several months, and repeatedly during this period the iodide of mercury was detected in the urine by chemical tests.

The great efficacy of the iodide of potassium as an antidote to the slow poison of lead and mercury, were proved by M. Melsens, by experiments upon several dogs, which were fed with the carbonate and sulphate of lead till paralyzed, emaciated, and nearly dead, and then in a short time restored to health and flesh by the administration of the iodide of potassium.

Three cases of severe lead paralysis among house-painters and workers in lead, were entirely cured, and a fourth greatly relieved, by the same remedy. In five cases of mercurial paralysis and severe suffering among gilders and workers in quicksilver, the iodide of potassium, in a few weeks, accomplished great relief, or a perfect cure.

It happened in some cases that the poison seemed to be liberated so rapidly by the remedy that it was badly borne. Sometimes profuse re-salivation was the consequence. I had under my own care a gilder, aged 65, a few weeks since, suffering from mercurial tremors and paralysis, in whom eight grains of the iodide of potassium, three times a day, produced distressing ptyalism, and so added to his sufferings that he refused to continue the remedy.

Does the iodide of potassium ever salivate, except by *liberating* mercury?

We believe not. In the few cases in which salivation occurs from preparations, of iodine, we think it will always be discovered that mercurials have, at some previous time in the patient's life, been taken.

Frem the experiments of M. Melsens, it appears that the iodide of potassium, when taken with mercurials, sometimes acts as a preventive to injury from the latter. We have for years been in the habit in strumous syphilitic cases, of giving blue pill at night, and the iodide of potassium by day. Or we have *neutralized*, as we imagined, the too severe effects of the prot. iodide of mercury, in syphilitic and scrofulous, throat affections, by combining with it the iodide of potassium. We have very lately witnessed excellent effects from this combination, in a case of tubercular syphilitic eruption.

Might not the exhibition of the iodide of potassium, in seasons of special exposure, be a protection to painters and workmen in lead, against lead colic and paralysis?

It is curious to notice what immense quantities of the iodide of potassium may be often safely borne. M. Melsens took, himself, for two months, from half a drachm to a drachm and a half per day, or more than two thousand grains in the whole period, without any inconvenience, except temporary coryza, and a few pimples, and with a decided increase of appetite. One of the most severe cases of mercurial paralysis, related as cured by him, took 2,314 grains of the iodide of potassium, between the 21st of March and the 23d of June.

Of the cases of our own, given above, No. 2 took five grains. three times a day, for eleven months, with the greatest benefit. M. Melsens recommends, in cases of mercurial, or lead poisoning, to begin with fifteen grains in solution, three times a day, and to increase the dose as the patient will bear it.

Dr. Budd thinks such large doses require two conditions—*First*, to be given on an empty stomach; and *secondly*, in a state of large dilution.

The cases we have narrated above, seem to prove that in milder forms, where mercurial paralysis is not induced, and the system is not highly charged with the noxious mineral, smaller doses of the iodide of potassium, if continued sufficiently long, are highly efficacious.

In conclusion, these researches of M. Melsens explain, we think, why the iodide of potassium is so serviceable in certain broken-down syphilitic patients, in whom the quantity of mercurials previously taken, finally form an important element in their disease. We have not sufficiently tested the iodide of potassium in lead disease, to speak as yet with confidence from personal reference. We may, however, as a supplement to this paper, at a future period, report a few cases of lead paralysis, now under treatment, and hope, in the mean time, to promote the principal object of this paper, by eliciting from the profession further observations on this important subject, and exciting a deeper interest in the original memoir, from which we have so freely quoted.—*New-York Journal of Medicine.*

